# Efficacy of Immune Checkpoint Inhibitor With or Without Chemotherapy for Nonsquamous NSCLC With Malignant Pleural Effusion: A Retrospective Multicenter Cohort Study

**DOI:** 10.1016/j.jtocrr.2022.100355

**Published:** 2022-06-03

**Authors:** Hayato Kawachi, Motohiro Tamiya, Yoshihiko Taniguchi, Toshihide Yokoyama, Shinya Yokoe, Yuko Oya, Mihoko Imaji, Fukuko Okabe, Masaki Kanazu, Yoshihiko Sakata, Shinya Uematsu, Satoshi Tanaka, Daisuke Arai, Go Saito, Hiroshi Kobe, Eisaku Miyauchi, Asuka Okada, Satoshi Hara, Toru Kumagai

**Affiliations:** a Department of Thoracic Oncology, Osaka International Cancer Institute, Osaka, Osaka, Japan; b Department of Pulmonary Medicine, Graduate School of Medical Science, Kyoto Prefectural University of Medicine, Kyoto, Kyoto, Japan; c Department of Internal Medicine, National Hospital Organization Kinki-Chuo Chest Medical Center, Sakai, Osaka, Japan; d Department of Respiratory Medicine, Kurashiki Central Hospital, Kurashiki, Okayama, Japan; e Department of Thoracic Oncology, Aichi Cancer Center Hospital, Nagoya, Aichi, Japan; f Internal Medicine III, Wakayama Medical University, Wakayama, Wakayama, Japan; g Department of Thoracic Oncology, National Hospital Organization Osaka Toneyama Medical Center, Toyonaka, Osaka, Japan; h Division of Respiratory Medicine, Saiseikai Kumamoto Hospital, Kumamoto, Kumamoto, Japan; i Department of Respiratory Medicine, Osaka Red Cross Hospital, Osaka, Osaka, Japan; j Department of Respiratory Medicine, Osaka General Medical Center, Osaka, Osaka, Japan; k Department of Internal Medicine, Saiseikai Utsunomiya Hospital, Utsunomiya, Tochigi, Japan; l Department of Respirology, Graduate School of Medicine, Chiba University, Chiba, Chiba, Japan; m Department of Respiratory Medicine, Kobe City Medical Center General Hospital, Kobe, Japan; n Department of Respiratory Medicine, Tohoku University Hospital, Sendai, Miyagi, Japan; o Department of Respiratory Medicine, Saiseikai Suita Hospital, Suita, Osaka, Japan; p Department of Respiratory Medicine, Itami City Hospital, Itami, Japan

**Keywords:** Immune checkpoint inhibitor, Combination therapy, Non–small cell lung cancer, Malignant pleural effusion, Treatment outcome

## Abstract

**Introduction:**

Malignant pleural effusion (MPE) is associated with poor treatment outcome in patients with NSCLC receiving immune checkpoint inhibitors (ICIs). ICIs and chemotherapy (ICI/Chemo) combination therapy is currently the standard therapy for NSCLC, and some ICI/Chemo regimens for nonsquamous (non-Sq) NSCLC contain bevacizumab (BEV), which is effective for controlling MPE and may enhance immune response. This study aimed to determine the optimal first-line treatment for this clinical population.

**Methods:**

We retrospectively enrolled consecutive patients with non-Sq NSCLC with MPE who received ICI/Chemo or pembrolizumab monotherapy. Treatment outcomes were analyzed in patients with programmed death-ligand 1 (PD-L1) tumor proportion score more than or equal to 50% who were administered ICI/Chemo or pembrolizumab monotherapy (PD-L1 high cohort) and in patients with any PD-L1 status, treated with ICI/Chemo with or without BEV (ICI/Chemo cohort). We used propensity score matching (PSM) to reduce bias.

**Results:**

PD-L1 high and ICI/Chemo cohorts included 143 and 139 patients, respectively. In PD-L1 high cohort, 37 patients received ICI/Chemo. With PSM, the median progression-free survival was significantly longer in the ICI/Chemo group than in the pembrolizumab group (11.1 versus 3.9 mo, respectively, *p* = 0.0409). In the ICI/Chemo cohort, 23 patients received BEV. With PSM, no significant difference occurred in median progression-free survival between BEV and non-BEV groups (6.1 versus 7.4 mo, *p* = 0.9610).

**Conclusion:**

ICI/Chemo seemed more effective than pembrolizumab monotherapy for patients with non-Sq NSCLC with MPE. Nevertheless, the synergistic effect of BEV with ICI/Chemo may be limited. Further studies are needed to clarify the key factor in the tumor-induced immunosuppression environment in these patients.

## Introduction

Lung cancer is a major cause of cancer-related death worldwide.^1^ NSCLC accounts for approximately 80% of lung cancer cases, and most NSCLC cases are diagnosed at advanced, unresectable, and metastatic disease stages.^2^ Immune checkpoint inhibitors (ICIs), such as programmed cell death protein 1/programmed death-ligand 1 (PD-L1) axis inhibitors, were found to have outstanding efficacy in many types of malignant neoplasms, including NSCLC.^3^, ^4^, ^5^, ^6^, ^7^, ^8^, ^9^ Particularly, pembrolizumab monotherapy is one of the standard first-line treatment options for patients with advanced NSCLC and PD-L1 tumor proportion score (TPS) more than or equal to 50%.^6^^,^^7^ Although higher PD-L1 TPS is associated with better treatment outcomes with ICIs, approximately 30% of patients with NSCLC experience disease progression after first-line pembrolizumab treatment, even with PD-L1 TPS more than or equal to 50%.^6^^,^^7^ Therefore, PD-L1 expression alone is insufficient as a predictive biomarker, and another useful predictive factor that enables easy optimal patient selection for ICI treatment in clinical settings is warranted.

We previously reported association between metastatic sites and treatment outcomes of first-line pembrolizumab for advanced NSCLC with a PD-L1 TPS more than or equal to 50%.^10^ We found that malignant pleural effusion (MPE) was considerably correlated with a shorter progression-free survival (PFS). Thus, for patients with NSCLC with MPE, pembrolizumab monotherapy is not a reasonable first-line treatment option because of its insufficient effectiveness, even though their PD-L1 TPS is high. MPE, which occurs in approximately 15% of patients with NSCLC, influences their management and quality of life^11^; therefore, a novel treatment strategy is urgently needed. Several phase 3 clinical trials for advanced NSCLC have recently reported that patients treated with a combination of ICI and chemotherapy (ICI/Chemo) have notably better clinical course than those treated with chemotherapy alone.^12^, ^13^, ^14^, ^15^, ^16^, ^17^ On the basis of these results, combination therapy has been established as a standard therapy in patients with treatment-naive advanced NSCLC without driver oncogenes, irrespective of the PD-L1 TPS.

With the immunogenic effects of cytotoxic chemotherapy, modulation of the immune response through ICIs is considered to be enhanced.^18^ Therefore, ICI/Chemo is considered a more effective treatment option than ICI monotherapy for patients with MPE. In addition, some ICI/Chemo regimens for nonsquamous (non-Sq) NSCLC contain bevacizumab (BEV), an antivascular endothelial growth factor (VEGF)-A agent.^14^^,^^17^ Inhibition of VEGF activity with BEV is effective in the management of MPE.^19^, ^20^, ^21^ Moreover, preclinical studies have revealed that BEV improves the efficacy of ICIs by blocking VEGF-mediated immunosuppression.^22^, ^23^, ^24^ On the basis of these findings, ICI/Chemo with BEV may be the optimal treatment option for patients with MPE. Thus, this study aimed to determine the optimal first-line treatment for patients with non-Sq NSCLC with MPE.

## Materials and Methods

### Study Design and Patients

This retrospective multicenter cohort study was conducted at 15 member institutions of Hanshin Oncology Clinical Problem Evaluation (HOPE) group in Japan. We included consecutive patients with non-Sq NSCLC with MPE who received pembrolizumab monotherapy or a combination therapy of ICIs plus chemotherapy as first-line therapy between March 2017 and September 2020. Patients with sensitizing *EGFR* mutation or *ALK* fusion were excluded. Clinical data at the time of first-line treatment initiation were collected from electronic medical records. Patients with a diagnosis of malignant pleural effusion with imaging and clinical evidence were considered eligible, even without cytology. This study was approved by the review boards of each of the 15 institutions. The requirement of informed consent was waived owing to the retrospective nature of the study.

### Assessments

The maximum volume of the malignant pleural effusion was assessed on computed tomography scans and classified into the following two categories: small (<10 mm thick) or large (≥10 mm thick).^22^ PD-L1 TPS in tumor cells was analyzed using PD-L1 immunohistochemistry 22C3 pharmDx antibody (clone 22C3; Dako North America, Inc., Carpinteria, CA). Treatment outcomes were assessed in patients with PD-L1 TPS more than or equal to 50% and compared between those with pembrolizumab monotherapy and ICI/Chemo (PD-L1 high cohort). They were also analyzed in patients with any PD-L1 TPS treated with ICI/Chemo (ICI/Chemo cohort) and compared between patients with and without BEV.

Treatment response was evaluated according to the Response Evaluation Criteria in Solid Tumors version 1.1.^25^ The incidence of adverse events was assessed according to the Common Terminology Criteria for Adverse Events version 5.0.^26^ PFS was measured from the start of first-line treatment to the first instance of lung cancer progression or death from any-cause death. Overall survival (OS) was measured from the start of first-line treatment to death from any-cause death. PFS of MPE was measured from the start of first-line treatment to reaccumulation of MPE, defined as the need for drainage or unequivocal increase compared with the baseline MPE on a chest radiograph or computed tomography scan. The data cutoff date was November 30, 2020.

### Statistical Analysis

Age was analyzed using the Wilcoxon ranked sum test. Dichotomous variables were analyzed using the chi-square test or Fisher’s exact test, as appropriate. Survival outcomes were estimated using the Kaplan–Meier method and compared using the log-rank test. We performed rigorous adjustment for significant differences in the baseline characteristics of patients using propensity score matching (PSM) including the following variables: age, sex, smoking status, Eastern Cooperative Oncology Group performance status, histologic diagnosis, level of PD-L1 TPS, MPE diagnosis by cytology, MPE volume, intervention for MPE, pleurodesis, liver metastasis, and brain metastasis. Nearest-neighbor matching was performed at a ratio of 1:1, without replacement. Caliper was set at 0.2. All analyses were performed using JMP 14 software (SAS Institute, Cary, NC). Statistical significance was defined as a two-tailed *p* value of less than 0.05.

## Results

### Patient Inclusion

**Figure 1:**
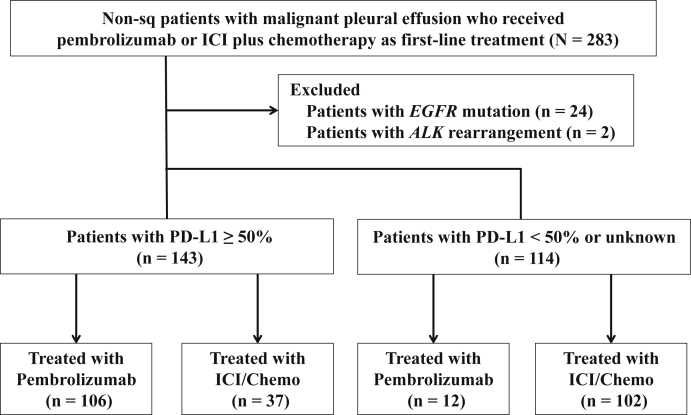
Flowchart for the study patients. ICI/Chemo, immune checkpoint inhibitor and chemotherapy; Non-sq, nonsquamous; PD-L1, programmed death-ligand 1.

Among the 283 patients with consecutive non-Sq NSCLC with MPE, 257 patients were evaluated (Fig. 1). Of the 257 patients, 143 patients had PD-L1 TPS more than or equal to 50%, of whom 106 received pembrolizumab monotherapy and 37 received ICI/Chemo. Whereas, of the 114 patients with PD-L1 TPS less than 50% or unknown, 12 received pembrolizumab monotherapy and 102 received ICI/Chemo. Overall, the PD-L1 high and ICI/Chemo cohorts included 143 and 139 patients, respectively.

### PD-L1 High Cohort

#### Patient Characteristics

The baseline characteristics of the PD-L1 high cohort (n = 143) are summarized in Supplementary Table 1. The median age was 72 (range: 39–89) years, and most patients were men (79%), had a smoking history (82%), had a performance status of 0 or 1 (73%), and had adenocarcinoma histology (87%). The PD-L1 TPS was 50% to 74% and 75% to 100% in 39% and 61% of the patients, respectively. In total, 52% of the patients were diagnosed with having MPE on the basis of cytologic examination of the pleural fluid. Furthermore, 28% of the patients had a small amount of MPE. Thoracentesis and chest tube drainage were performed in 28% and 31% of the patients, respectively. In addition, 23% of the patients underwent pleurodesis for the management of MPE.

In the ICI/Chemo group, a pemetrexed-containing regimen was administered to 78% of the patients, whereas BEV was administered to 16% of the patients. Compared with the ICI/Chemo group, the pembrolizumab group had significantly higher proportions of older patients (72 [range: 39–89] y versus 69 [range: 46–79 y], respectively, *p* = 0.057), patients aged more than or equal to 75 years (58/106 versus 31/84, *p* = 0.0017), and male patients (89/106 versus 25/37, respectively, *p* = 0.0327). After PSM, 32 patients were included in each group. There were no significant differences in baseline characteristics between the two groups after matching (Table 1).

**Table 1:** Patient Characteristics in PD-L1 High Cohort and Comparison Between Pembrolizumab Group and ICI Plus Chemotherapy Group Adjusted by Propensity Score Matching (N = 64)

Patient Characteristics	All Patients (N = 64)	Pembrolizumab Group (n = 32)	ICI Plus Chemotherapy Group (n = 32)	*p* Value
Age (y)				
Median (range)	69.5 (46-81)	69.5 (48-81)	69.5 (46-79)	0.6331
<75 y	52 (81)	26 (81)	26 (81)	1.0000
≥75 y	12 (19)	6 (19)	6 (19)	
Sex				
Male	44 (69)	22 (69)	22 (69)	1.0000
Female	20 (31)	10 (31)	10 (31)	
Smoking status				
Never smoker	14 (22)	7 (22)	7 (22)	1.0000
Current or former smoker	50 (78)	25 (78)	25 (78)	
ECOG PS				
0–1	46 (72)	22 (68)	24 (75)	0.5782
2–4	18 (28)	10 (31)	8 (25)	
Histologic diagnosis				
Adenocarcinoma	57 (89)	28 (88)	29 (91)	0.6888
Other	7 (11)	4 (13)	3 (9)	
PD-L1 status				
50%–74%	23 (36)	11 (34)	12 (38)	0.7945
75%–100%	41 (64)	21 (66)	20 (62)	
Pleural fluid cytology				
Confirmed	31 (48)	15 (47)	16 (50)	0.8025
Volume of malignant pleural effusion				
Small	16 (25)	7 (22)	9 (28)	0.5637
Large	48 (75)	25 (78)	23 (72)	
Pleural intervention				
Not performed	27 (42)	13 (41)	14 (44)	0.8459
Thoracentesis	21 (33)	10 (31)	11 (34)	
Chest tube drainage	16 (25)	9 (28)	7 (22)	
Pleurodesis				
Performed	11 (17)	6 (19)	5 (16)	0.7404
Metastatic site				
Liver metastasis	4 (6)	1 (3)	3 (9)	0.3017
Brain metastasis	8 (13)	3 (9)	5 (16)	0.4497
Bone metastasis	22 (34)	12 (38)	10 (31)	0.5986
Adrenal metastasis	7 (11)	3 (9)	4 (13)	0.6888
Treatment regimen				
Pembrolizumab	32 (74)	32 (100)		
CBDCA/PEM/pembrolizumab	20 (31)		20 (63)	
CDDP/PEM/pembrolizumab	3 (5)		3 (9)	
CBDCA/PEM/atezolizumab	2 (4)		2 (6)	
CBDCA/PTX/BEV/atezolizumab	5 (8)		5 (16)	
CBDCA/nab-PTX/atezolizumab	2 (3)		2 (6)	

BEV, bevacizumab; CBDCA, carboplatin; CDDP, cisplatin; ECOG PS, Eastern Cooperative Oncology Group performance status; ICI, immune checkpoint inhibitor; nab-PTX, nanoparticle albumin-bound paclitaxel; PD-L1, programmed death death-ligand 1; PEM, pemetrexed; PTX, paclitaxel.

#### Treatment Outcomes

The objective response rate (ORR) and disease control rate (DCR) were significantly higher in the ICI/Chemo group than in the pembrolizumab group (80.0% versus 41.2%, *p* = 0.0001; 94.3% versus 51.8%, *p* < 0.0001). The median length of follow-up was 11.0 months for all patients in the PD-L1 high cohort. Both the median PFS (11.1 mo versus 3.1 mo, *p* = 0.0049) (Supplementary Fig. 1*A*) and the median OS (22.7 mo versus 14.9 mo, *p* = 0.0156) (Supplementary Fig. 1*B*) were significantly longer in the ICI/Chemo group than in the pembrolizumab group. There were no significant between-group differences in the median PFS of MPE between the pembrolizumab and the ICI/Chemo groups. At 8 weeks after the administration of the first-line treatment, the DCR of MPE was 91.7% in the ICI/Chemo group and 70.7% in the pembrolizumab group.

**Figure 2:**
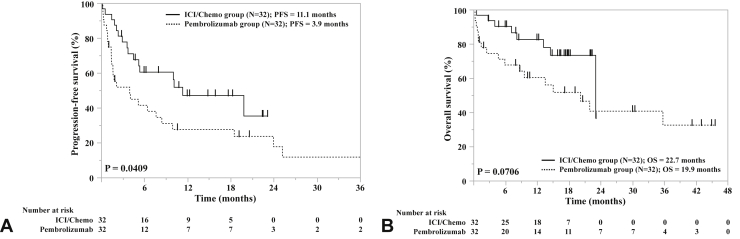
Kaplan–Meier survival curves revealing the progression-free survival (*A*) and overall survival (*B*) in PD-L1 high cohort after PSM. ICI/Chemo, immune checkpoint inhibitor and chemotherapy; PD-L1, programmed death-ligand 1; PFS, progression-free survival; PSM, propensity score matching.

Analysis of the treatment outcomes after adjusting for PSM revealed that the ORR and DCR were significantly higher in the ICI/Chemo group than in the pembrolizumab group (76.7% versus 34.6%, *p* = 0.0015 and 93.3% versus 50.0%, *p* = 0.0003, respectively). The median PFS was also significantly longer in the ICI/Chemo group than in the pembrolizumab group (11.1 mo versus 3.9 mo, *p* = 0.0409) (Fig. 2*A*). The median OS was longer in the ICI/Chemo group than in the pembrolizumab group, but the difference did not reach statistical significance (22.7 mo versus 19.9 mo, *p* = 0.0706) (Fig. 2*B*). There were no significant differences in the PFS of MPE between the pembrolizumab and ICI/Chemo groups. At 8 weeks after the administration of the first-line treatment, the DCR of MPE was 90.3% in the ICI/Chemo group and 81.9% in the pembrolizumab group.

#### Safety

The incidence of grade more than or equal to three drug-related adverse events in PD-L1 high cohort is summarized in Supplementary Table 2. Overall, 34% (49 of 143) of patients in PD-L1 high cohort developed adverse events of grade more than or equal to 3, 28% (30 of 106) of patients in the pembrolizumab group, and 51% (19 of 37) of patients in the ICI/Chemo group. In the pembrolizumab group, 15% (16 of 106) and 10% (11 of 106) of the patients developed any grade of pneumonitis and more than or equal to grade 3 of pneumonitis, respectively. Whereas, 27% (10 of 37) and 14% (5 of 37) of the patients developed any grade of pneumonitis and more than or equal to grade 3 of pneumonitis in the ICI/Chemo group, respectively.

### ICI/Chemo Cohort

#### Patient Characteristics

The baseline characteristics of the ICI/Chemo cohort (n = 139) are summarized in Supplementary Table 3. The median age was 69 (range: 44–84) years, and most patients were men (75%), had a smoking history (81%), had a performance status of 0 or 1 (85%), and had adenocarcinoma histology (91%). The PD-L1 TPS was 0%, 1% to 49%, 50% to 74%, 75% to 100%, and unknown in 33%, 29%, 9%, 17%, and 12%, respectively. A total of 50% of the patients were diagnosed with having MPE, and 27% of the patients had a small amount of MPE. Thoracentesis and chest tube drainage were performed in 27% and 26% of the patients, respectively. Furthermore, 17% of the patients underwent pleurodesis for the management of MPE. BEV was administered in 23 patients (17%). Compared with the non-BEV group, the BEV group had a significantly higher proportion of male patients (22 of 23 versus 82 of 116, *p* = 0.0117). After PSM, 21 patients were included in each group. There were no significant differences in baseline characteristics between the two groups after matching (Table 2).

**Table 2:** Patient Characteristics in ICI/Chemo Cohort and Comparison Between Bevacizumab and Non-Bevacizumab Group Adjusted by Propensity Score Matching (N = 42)

Patient Characteristics	All Patients (N = 42)	With Bevacizumab Group (n = 21)	Without Bevacizumab Group (n = 21)	*p* Value
Age (y)				
Median (range)	68.5 (44-79)	69 (51-79)	67 (44-78)	0.3643
<75 y	35 (83)	16 (76)	19 (90)	0.2142
≥75 y	7 (17)	5 (24)	2 (10)	
Sex				
Male	41 (98)	20 (95)	21 (100)	0.3115
Female	1 (2)	1 (5)	0 (0)	
Smoking status				
Never smoker	3 (7)	2 (10)	1 (5)	0.5491
Current or former smoker	39 (93)	19 (90)	20 (95)	
ECOG PS				
0–1	33 (79)	18 (86)	15 (71)	0.2593
2–4	9 (21)	3 (14)	6 (29)	
Histologic diagnosis				
Adenocarcinoma	39 (93)	19 (90)	20 (95)	0.5491
Other	3 (7)	2 (10)	1 (5)	
PD-L1 status				
0%	10 (24)	6 (29)	4 (19)	0.9352
1%–49%	16 (38)	8 (38)	8 (38)	
50%–74%	3 (7)	1 (5)	2 (10)	
75%–100%	11 (26)	5 (24)	6 (29)	
Unknown	2 (5)	1 (5)	1 (5)	
Pleural fluid cytology				
Confirmed	24 (57)	12 (57)	12 (57)	1.0000
Volume of malignant pleural effusion				
Small	8 (19)	4 (19)	4 (19)	1.0000
Large	34 (81)	17 (81)	17 (81)	
Pleural intervention				
Not performed	18 (43)	9 (43)	9 (43)	1.0000
Thoracentesis	8 (19)	4 (19)	4 (19)	
Chest tube drainage	16 (38)	8 (38)	8 (38)	
Pleurodesis				
Performed	4 (10)	2 (10)	2 (10)	1.0000
Metastatic site				
Liver metastasis	6 (14)	3 (14)	3 (14)	1.0000
Brain metastasis	6 (14)	3 (14)	3 (14)	1.0000
Bone metastasis	7 (17)	5 (24)	2 (10)	0.2142
Adrenal metastasis	4 (10)	1 (5)	3 (14)	0.2931
Treatment regimen				
CBDCA/PEM/pembrolizumab	15 (36)		15 (71)	
CDDP/PEM/pembrolizumab	3 (7)		3 (14)	
CBDCA/PEM/atezolizumab	1 (2)		1 (5)	
CDDP/PEM/atezolizumab	2 (5)		2 (10)	
CBDCA/PTX/BEV/atezolizumab	21 (50)	21 (100)	0 (0)	
CBDCA/nab-PTX/atezolizumab	0 (0)		0 (0)	

BEV, bevacizumab; CBDCA, carboplatin; CDDP, cisplatin; Chemo, chemotherapy; ECOG PS, Eastern Cooperative Oncology Group performance status; ICI, immune checkpoint inhibitor; nab-PTX, nanoparticle albumin-bound paclitaxel; PD-L1, programmed death-ligand 1; PEM, pemetrexed; PTX, paclitaxel.

#### Treatment Outcomes

The ORR was significantly higher in the BEV group than in the non-BEV group (80.0% versus 45.5%, *p* = 0.0049). Meanwhile, although the DCR was higher in the BEV group than in the non-BEV group, the difference was not significant (90.0% versus 80.2%, *p* = 0.2991). The median length of follow-up was 10.1 months for all patients in the ICI/Chemo cohort. There were also no significant between-group differences in the median PFS between the BEV and non-BEV groups (5.7 mo versus 6.0 mo, *p* = 0.4663) (Supplementary Fig. 2). There were no significant differences in the PFS of MPE between the BEV and non-BEV groups. At 8 weeks after the administration of first-line treatment, the DCR of MPM was 95.5% in the BEV group and 89.1% in the non-BEV group.

**Figure 3:**
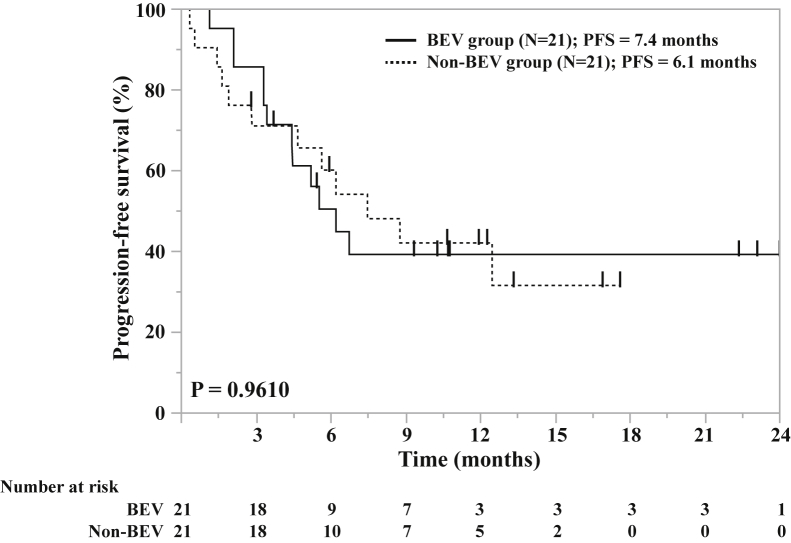
Kaplan–Meier survival curves revealing the progression-free survival in ICI/Chemo cohort after PSM. BEV, bevacizumab; ICI/Chemo, immune checkpoint inhibitor and chemotherapy; PFS, progression-free survival; PSM, propensity score matching.

Analysis of treatment outcomes after PSM revealed no significant difference in the ORR (79.0% versus 50.0%, *p* = 0.0653) and DCR (89.5% versus 88.9%, *p* = 0.9543) between the BEV and non-BEV groups. There were also no significant differences in the median PFS between the BEV and non-BEV groups (6.1 mo versus 7.4 mo, *p* = 0.9610) (Fig. 3). There were no significant differences in the PFS of MPE between the BEV and non-BEV groups. At 8 weeks after the administration of the first-line treatment, the DCR of MPE was 95.0% in the BEV group and 95.0% in the non-BEV group.

#### Safety

The incidence of grade more than or equal to three drug-related adverse events in the ICI/Chemo cohort is summarized in Supplementary Table 4. Overall, those who developed adverse events of more than or equal to grade 3 were 52% (72 of 139) of the patients in the ICI/Chemo cohort, 70% (16 of 23) of the patients in the BEV group, and 48% (56 of 116) of the patients in the non-BEV group. In the BEV group, 13% (3 of 23) and 4% (1 of 23) of the patients developed any grade of pneumonitis and more than or equal to grade 3 of pneumonitis, respectively. Whereas, 19% (22 of 116) and 7% (8 of 116) of the patients developed any grade of pneumonitis and more than or equal to grade 3 of pneumonitis in the non-BEV group, respectively.

## Discussion

The results of the present study revealed the treatment benefit of the combination of ICIs and chemotherapy for patients with PD-L1 high non-Sq NSCLC with MPE. Furthermore, the synergistic effect of BEV and ICI/Chemo may be limited. To the best of our knowledge, this is the first study to analyze the treatment outcomes of ICI/Chemo in patients with MPE.

In a preclinical study, cytotoxic chemotherapy enhanced anticancer immunity through programmed cell death protein-1 inhibition, such as the release of potentially immunogenic tumor antigens induced by tumor cell death.^18^ On the basis of these findings, ICI/Chemo may be clinically more effective than pembrolizumab monotherapy, although it is more toxic than pembrolizumab monotherapy. Therefore, both ICI/Chemo and pembrolizumab monotherapy are valid treatment options for patients with PD-L1 high, and predictive biomarkers are needed to inform clinical decisions to select the optimal treatment. We conducted this study based on a previous report that the presence of MPE is a predictive factor for poor treatment outcomes with pembrolizumab.^10^ The results confirmed that MPE is a useful and easily assessable factor for the administration of combination therapy. Considering the treatment strategy in patients with PD-L1 high NSCLC, a negative biomarker for ICI monotherapy may also be useful for the administration of ICI/Chemo. Further studies are required to clarify these associations.

In a preclinical study, inhibition of VEGF caused transient vascular normalization, leading to reduced tumor hypoxia and increased immune cell infiltration, which may result in increased antitumor immunity.^22^, ^23^, ^24^ On the basis of these findings, ICIs plus VEGF inhibitors are considered effective combination treatment options. A combination of atezolizumab and BEV revealed favorable ORR and PFS in patients with non-Sq NSCLC with PD-L1 high expression in a phase 2 clinical trial.^27^ This clinical result supports the benefit of adding BEV to ICI monotherapy. A randomized phase 3 trial for patients with non-Sq NSCLC has been conducted to better clarify the effect of adding BEV to ICI plus chemotherapy,^28^ and the results are awaited to address this clinically important issue.

Notably, our study revealed the role of VEGF in patients with non-Sq NSCLC with MPE receiving ICI/Chemo, which could be a novel finding for this patient population. VEGF plays a major role in causing MPE by increasing vascular and mesothelial permeability and capillary fluid leakage.^29^ Furthermore, VEGF is considered a key factor in tumor-induced immunosuppression.^22^, ^23^, ^24^ Nevertheless, in the result of our study, adding BEV did not translate into an advantage, which indicates that the addition of BEV may not be associated with improvement of durable antitumor response for non-Sq tumors with MPE treated with ICI/Chemo. It is possible that VEGF was not an independent factor for poor treatment outcomes after ICI/Chemo, and other cytokines may be associated with MPE. MPE contains multiple innate immune cells, and these immune cells release cytokines, including VEGF, IL-6, IL-8, and tumor growth factor β, that suppress the effectiveness of immune checkpoint inhibition.^30^, ^31^, ^32^, ^33^ Thus, various cytokines are associated with the immunosuppressive environment status in patients with MPE, and VEGF blockade alone may be insufficient to enhance the immune response. Considering the optimal treatment strategy, further studies are needed to clarify the factors that are independently associated with the immunosuppressive environment in patients with MPE.

The present study had some limitations. First, this study had a multicenter retrospective design. Therefore, the possibility of selection bias could not be ruled out. Nevertheless, the patients were consecutively enrolled, and PSM was conducted to reduce bias. Second, the study included only Japanese patients, which precludes the generalizability of our findings to patients from other countries. Third, regarding analysis of OS, the number of events was limited in each treatment group. Therefore, the observation period may not be long enough to investigate the long-term survival outcome. Fourth, in the ICI/Cohort, the sample size of the BEV group was small. In addition, there were differences regarding combination regimens between the BEV and non-BEV groups, and the effect of this difference on treatment outcome is inevitable. On the basis of these limitations, although the results of our study are clinically important, it may not lead to firm conclusion. To confirm our findings, further studies with large cohorts are warranted.

In conclusion, ICI/Chemo is a more effective treatment option than pembrolizumab monotherapy, even for patients with NSCLC with PD-L1 TPS more than or equal to 50%. The presence of MPE is a useful and simple predictive clinical factor to be considered in treatment decision making. Nevertheless, the synergistic effect of BEV and ICI/Chemo may be limited. Further studies are needed to clarify a key factor in the tumor-induced immunosuppression environment in these patient populations.

## CRediT Authorship Contribution Statement

**Hayato Kawachi**: Conceptualization, Methodology, Formal analysis, Investigation, Resources, Data curation, Writing—original draft, Writing—review and editing, Visualization, Supervision, and Project administration.

**Motohiro Tamiya**: Conceptualization, Methodology, Investigation, Resources, Data curation, Writing—review and editing, and Project administration.

**Yoshihiko Taniguchi, Toshihide Yokoyama, Shinya Yokoe, Yuko Oya, Mihoko Imaji, Fukuko Okabe, Masaki Kanazu, Yoshihiko Sakata, Shinya Uematsu, Satoshi Tanaka, Daisuke Arai, Go Saito, Hiroshi Kobe, Eisaku Miyauchi, Asuka Okada, Satoshi Hara, Toru Kumagai**: Investigation, Resources, and Writing—review and editing.

## Data Availability

The data sets generated during the current study are available from the corresponding author on reasonable request.

## Ethics Approval

All procedures performed in studies involving human participants were in accordance with the ethical standards of the institutional and/or national research committee and with the 1964 Helsinki declaration and its later amendments or comparable ethical standards. The study protocol was approved by the review board of each of the four institutions.

## Informed Consent

The requirement for the patients’ informed consent was waived owing to the retrospective nature of the study, and an opt-out method was included so that patients and families could opt-out of participating in the study.

## Supplementary Data

Supplementary Table1

Supplementary Table2

Supplementary Table3

Supplementary Table4

Supplementary Figure1A

Supplementary Figure1B

Supplementary Figure2
