# Supplementary material for: Efficacy of Immune Checkpoint Inhibitor With or Without Chemotherapy for Nonsquamous NSCLC With Malignant Pleural Effusion: A Retrospective Multicenter Cohort Study
Source: JTO Clin Res Rep. 2022 Jun 3;3(7):100355. doi: 10.1016/j.jtocrr.2022.100355 (PMC9234704; doi:10.1016/j.jtocrr.2022.100355)
Supplement: Supplementary Table1 [file mmc1.docx]

**Supplementary Table 1.** Patient characteristics in PD-L1 high cohort and comparison between pembrolizumab group and ICI plus chemotherapy group (N = 143).

| **Patient characteristics** | **All patients**  **(N = 143)** | **Pembrolizumab group**  **(n = 106)** | **ICI plus Chemotherapy group**  **(n = 37)** | **P value** |
| --- | --- | --- | --- | --- |
| Age (years) |  |  |  |  |
| Median (range) | 72 (39-89) | 72 (39-89) | 69 (46-79) | 0.0057 |
| ＜75 years | 89 (62) | 58 (55) | 31 (84) | 0.0017 |
| ≧75 years | 54 (38) | 48 (45) | 6 (16) |  |
| Sex |  |  |  |  |
| Male | 114 (79) | 89 (84) | 25 (68) | 0.0327 |
| Female | 29 (20) | 17 (16) | 12 (32) |  |
| Smoking status |  |  |  |  |
| Never-smoker | 26 (18) | 18 (17) | 8 (22) | 0.5286 |
| Current or former smoker | 117 (82) | 88 (83) | 29 (78) |  |
| ECOG PS |  |  |  |  |
| 0-1 | 105 (73) | 77 (73) | 28 (76) | 0.7191 |
| 2-4 | 38 (27) | 29 (27) | 9 (24) |  |
| Histologic diagnosis |  |  |  |  |
| Adenocarcinoma | 124 (87) | 90 (85) | 34 (92) | 0.2811 |
| Other | 19 (13) | 16 (15) | 3 (8) |  |
| PD-L1 status |  |  |  |  |
| 50-74% | 56 (39) | 43 (41) | 13 (35) | 0.5601 |
| 75-100% | 87 (61) | 63 (59) | 24 (65) |  |
| Pleural fluid cytology |  |  |  |  |
| Confirmed | 74 (52) | 58 (55) | 16 (43) | 0.2292 |
| Volume of malignant pleural effusion |  |  |  |  |
| Small | 40 (28) | 31 (29) | 9 (24) | 0.5659 |
| Large | 103 (72) | 75 (71) | 28 (76) |  |
| Pleural intervention |  |  |  |  |
| Not performed | 59 (41) | 42 (40) | 17 (46) | 0.1773 |
| Thoracentesis | 40 (28) | 27 (26) | 13 (35) |  |
| Chest tube drainage | 44 (31) | 37 (35) | 7 (19) |  |
| Pleurodesis |  |  |  |  |
| Performed | 33 (23) | 28 (26) | 5 (14) | 0.1088 |
| Metastatic site |  |  |  |  |
| Liver metastasis | 13 (9) | 10 (9) | 3 (8) | 0.8091 |
| Brain metastasis | 20 (14) | 15 (14) | 5 (14) | 0.9233 |
| Bone metastasis | 49 (34) | 37 (35) | 12 (32) | 0.7849 |
| Adrenal metastasis | 22 (15) | 17 (16) | 5 (14) | 0.7141 |
| Treatment regimen |  |  |  |  |
| Pembrolizumab | 106 (74) | 106 (100) |  |  |
| CBDCA/PEM/Pembrolizumab | 24 (17) |  | 24 (65) |  |
| CDDP/PEM/Pembrolizumab | 3 (2) |  | 3 (8) |  |
| CBDCA/PEM/Atezolizumab | 2 (1) |  | 2 (5) |  |
| CBDCA/PTX/BEV/Atezolizumab | 2 (1) |  | 6 (16) |  |
| CBDCA/nab-PTX/ Atezolizumab | 6 (4) |  | 2 (5) |  |

ICI, immune checkpoint inhibitor; ECOG PS, Eastern Cooperative Oncology Group Performance Status; PD-L1, programmed death ligand 1; CBDCA, carboplatin; CDDP, cisplatin; PEM, pemetrexed; nab-PTX, nanoparticle albumin-bound paclitaxel; PTX, paclitaxel; BEV, bevacizumab.
