# Supplementary material for: Efficacy of Immune Checkpoint Inhibitor With or Without Chemotherapy for Nonsquamous NSCLC With Malignant Pleural Effusion: A Retrospective Multicenter Cohort Study
Source: JTO Clin Res Rep. 2022 Jun 3;3(7):100355. doi: 10.1016/j.jtocrr.2022.100355 (PMC9234704; doi:10.1016/j.jtocrr.2022.100355)
Supplement: Supplementary Table2 [file mmc2.docx]

**Supplementary Table2.** The incidences of drug-related adverse events of grade ≥ 3 in PD-L1 high cohort (=143)

|  | **All patients**  **(N=143)** | **Pembrolizumab group**  **(N=106)** | **ICI plus chemotherapy group**  **(N=37)** |
| --- | --- | --- | --- |
| Overall | 49 (34) | 30 (28) | 19 (51) |
| Pneumonitis | 16 (11) | 11 (10) | 5 (14) |
| Rash | 7 (5) | 5 (5) | 2 (5) |
| Infection | 6 (4) | 3 (3) | 3 (8) |
| Liver dysfunction | 6 (4) | 6 (6) | 0 (0) |
| Neutrophil count decreased | 6 (4) | 0 (0) | 6 (16) |
| White blood cell decreased | 4 (3) | 0 (0) | 4 (11) |
| Platelet count decreased | 2 (1) | 0 (0) | 2 (5) |
| Tumor lysis syndrome | 1 (1) | 1 (1) | 0 (0) |
| Encephalopathy | 1 (1) | 1 (1) | 0 (0) |
| Colitis | 1 (1) | 1 (1) | 0 (0) |
| Arthralgia | 1 (1) | 1 (1) | 0 (0) |
| Cholangitis | 1 (1) | 1 (1) | 0 (0) |
| Adrenal insufficiency | 1 (1) | 1 (1) | 0 (0) |
| Colonic perforation | 1 (1) | 1 (1) | 0 (0) |
| Mucositis oral | 1 (1) | 1 (1) | 0 (0) |
| Thromboembolic event | 1 (1) | 1 (1) | 0 (0) |
| Acute coronary syndrome | 1 (1) | 0 (0) | 1 (1) |
| Kidney dysfunction | 1 (1) | 0 (0) | 1 (1) |
| Pancreatitis | 1 (1) | 0 (0) | 1 (1) |
| Anemia | 1 (1) | 0 (0) | 1 (1) |
| Malaise | 1 (1) | 0 (0) | 1 (1) |
| Glaucoma | 1 (1) | 0 (0) | 1 (1) |
| Anorexia | 1 (1) | 0 (0) | 1 (1) |
