# Supplementary material for: Efficacy of Immune Checkpoint Inhibitor With or Without Chemotherapy for Nonsquamous NSCLC With Malignant Pleural Effusion: A Retrospective Multicenter Cohort Study
Source: JTO Clin Res Rep. 2022 Jun 3;3(7):100355. doi: 10.1016/j.jtocrr.2022.100355 (PMC9234704; doi:10.1016/j.jtocrr.2022.100355)
Supplement: Supplementary Table3 [file mmc3.docx]

**Supplementary Table 3.** Patient characteristics in ICI/Chemo cohort and comparison between bevacizumab and non-bevacizumab group (N = 139).

| **Patient characteristics** | **All patients**  **(N = 139)** | **Bevacizumab group**  **(n = 23)** | **Non-bevacizumab group**  **(n = 116)** | **P value** |
| --- | --- | --- | --- | --- |
| Age (years) |  |  |  |  |
| Median (range) | 69 (44-84) | 69 (51-79) | 69 (44-84) | 0.9412 |
| ＜75 years | 114 (82) | 18 (78) | 96 (83) | 0.6079 |
| ≧75 years | 25 (18) | 5 (22) | 20 (17) |  |
| Sex |  |  |  |  |
| Male | 104 (75) | 22 (96) | 82 (71) | 0.0117 |
| Female | 35 (25) | 1 (4) | 34 (29) |  |
| Smoking status |  |  |  |  |
| Never-smoker | 27 (19) | 4 (17) | 23 (20) | 0.7873 |
| Current or former smoker | 112 (81) | 19 (83) | 93 (80) |  |
| ECOG PS |  |  |  |  |
| 0-1 | 118 (85) | 20 (87) | 98 (84) | 0.7622 |
| 2-4 | 21 (15) | 3 (13) | 18 (16) |  |
| Histologic diagnosis |  |  |  |  |
| Adenocarcinoma | 127 (91) | 21 (91) | 106 (91) | 0.9907 |
| Other | 12 (9) | 2 (9) | 10 (9) |  |
| PD-L1 status |  |  |  |  |
| 0% | 46 (33) | 7 (30) | 39 (33) | 0.4977 |
| 1-49% | 40 (29) | 9 (39) | 31 (27) |  |
| 50-74% | 13 (9) | 1 (4) | 12 (10) |  |
| 75-100% | 24 (17) | 5 (22) | 19 (16) |  |
| unknown | 16 (12) | 1 (4) | 15 (13) |  |
| Pleural fluid cytology |  |  |  |  |
| confirmed | 69 (50) | 14 (61) | 55 (47) | 0.2384 |
| Volume of malignant pleural effusion |  |  |  |  |
| Small | 38 (27) | 4 (17) | 34 (29) | 0.2413 |
| Large | 101 (73) | 19 (83) | 82 (71) |  |
| Pleural intervention |  |  |  |  |
| Not performed | 66 (47) | 9 (39) | 57 (49) | 0.1015 |
| Thoracentesis | 37 (27) | 4 (17) | 33 (28) |  |
| Chest tube drainage | 36 (26) | 10 (43) | 26 (22) |  |
| Pleurodesis |  |  |  |  |
| Performed | 23 (17) | 4 (17) | 19 (16) | 0.9050 |
| Metastatic site |  |  |  |  |
| Liver metastasis | 12 (9) | 2 (9) | 10 (9) | 0.9907 |
| Brain metastasis | 17 (12) | 3 (13) | 14 (12) | 0.8963 |
| Bone metastasis | 46 (33) | 5 (22) | 41 (35) | 0.2052 |
| Adrenal metastasis | 19 (14) | 1 (4) | 18 (16) | 0.1543 |
| Treatment regimen |  |  |  |  |
| CBDCA/PEM/Pembrolizumab | 83 (60) |  | 83 (72) |  |
| CDDP/PEM/Pembrolizumab | 13 (9) |  | 13 (11) |  |
| CBDCA/PEM/Atezolizumab | 9 (6) |  | 9 (8) |  |
| CDDP/PEM/Atezolizumab | 2 (1) |  | 2 (2) |  |
| CBDCA/PTX/BEV/Atezolizumab | 23 (17) | 23 (100) |  |  |
| CBDCA/nab-PTX/ Atezolizumab | 9 (6) |  | 9 (8) |  |

ICI, immune checkpoint inhibitor; Chemo, chemotherapy; ECOG PS, Eastern Cooperative Oncology Group Performance Status; PD-L1, programmed death ligand 1; CBDCA, carboplatin; CDDP, cisplatin; PEM, pemetrexed; nab-PTX, nanoparticle albumin-bound paclitaxel; PTX, paclitaxel; BEV, bevacizumab.
