# Supplementary material for: Efficacy of Immune Checkpoint Inhibitor With or Without Chemotherapy for Nonsquamous NSCLC With Malignant Pleural Effusion: A Retrospective Multicenter Cohort Study
Source: JTO Clin Res Rep. 2022 Jun 3;3(7):100355. doi: 10.1016/j.jtocrr.2022.100355 (PMC9234704; doi:10.1016/j.jtocrr.2022.100355)
Supplement: Supplementary Table4 [file mmc4.docx]

**Supplementary Table4.** The incidences of drug-related adverse events of grade ≥ 3 in ICI/Chemo cohort (N=139).

|  | **Total**  **(N=139)** | **Bevacizumab group**  **(N=23)** | **Non-bevacizumab group**  **(N=116)** |
| --- | --- | --- | --- |
| Overall | 72 (52) | 16 (70) | 56 (48) |
| Neutrophil count decreased | 22 (15) | 6 (26) | 16 (14) |
| Platelet count decreased | 14 (10) | 3 (13) | 11 (9) |
| Anemia | 11 (8) | 1 (4) | 10 (9) |
| Pneumonitis | 9 (6) | 1 (4) | 8 (7) |
| Infection | 9 (6) | 2 (9) | 7 (6) |
| White blood cell decreased | 8 (6) | 2 (9) | 6 (5) |
| Febrile neutropenia | 6 (4) | 2 (9) | 4 (3) |
| Rash | 5 (4) | 1 (4) | 4 (3) |
| Liver dysfunction | 4 (3) | 1 (4) | 3 (3) |
| Anorexia | 4 (3) | 1 (4) | 3 (3) |
| Nausea | 4 (3) | 2 (9) | 2 (2) |
| Hyponatremia | 4 (3) | 1 (4) | 3 (3) |
| Adrenal insufficiency | 2 (1) | 0 (0) | 2 (2) |
| Kidney dysfunction | 2 (1) | 1 (4) | 1 (1) |
| Anaphylaxis | 2 (1) | 1 (4) | 1 (1) |
| Encephalopathy | 1 (1) | 0 (0) | 1 (1) |
| Acute coronary syndrome | 1 (1) | 0 (0) | 1 (1) |
| Pancreatitis | 1 (1) | 0 (0) | 1 (1) |
| Malaise | 1 (1) | 0 (0) | 1 (1) |
| Glaucoma | 1 (1) | 0 (0) | 1 (1) |
| Hypothyroidism | 1 (1) | 0 (0) | 1 (1) |
| Hip fracture | 1 (1) | 0 (0) | 1 (1) |
| Myasthenia gravis | 1 (1) | 0 (0) | 1 (1) |
