# Supplementary material for: Efficacy of Immune Checkpoint Inhibitor With or Without Chemotherapy for Nonsquamous NSCLC With Malignant Pleural Effusion: A Retrospective Multicenter Cohort Study
Source: JTO Clin Res Rep. 2022 Jun 3;3(7):100355. doi: 10.1016/j.jtocrr.2022.100355 (PMC9234704; doi:10.1016/j.jtocrr.2022.100355)
Supplement: Supplementary Figure1A [file mmc5.pptx]

## Slide 1
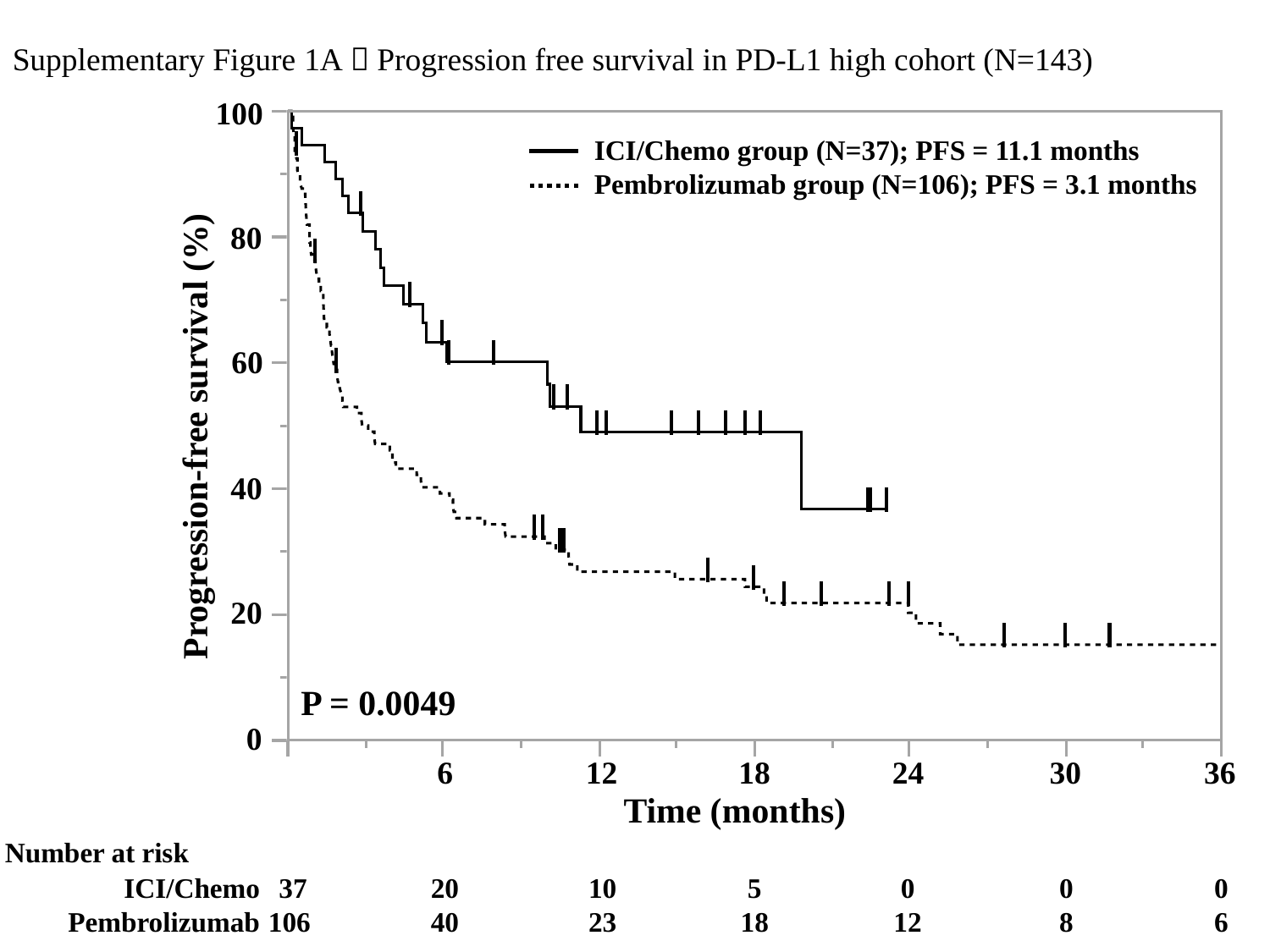

Supplementary Figure 1A：Progression free survival in PD-L1 high cohort (N=143)
100
ICI/Chemo group (N=37); PFS = 11.1 months
Pembrolizumab group (N=106); PFS = 3.1 months
Progression-free survival (%)
80
60
40
20
P = 0.0049
0
6
12
18
24
30
36
Time (months)
Number at risk
ICI/Chemo
Pembrolizumab
 37
106
20
40
10
23
5
18
0
12
0
8
0
6
