# Supplementary material for: Efficacy of Immune Checkpoint Inhibitor With or Without Chemotherapy for Nonsquamous NSCLC With Malignant Pleural Effusion: A Retrospective Multicenter Cohort Study
Source: JTO Clin Res Rep. 2022 Jun 3;3(7):100355. doi: 10.1016/j.jtocrr.2022.100355 (PMC9234704; doi:10.1016/j.jtocrr.2022.100355)
Supplement: Supplementary Figure1B [file mmc6.pptx]

## Slide 1
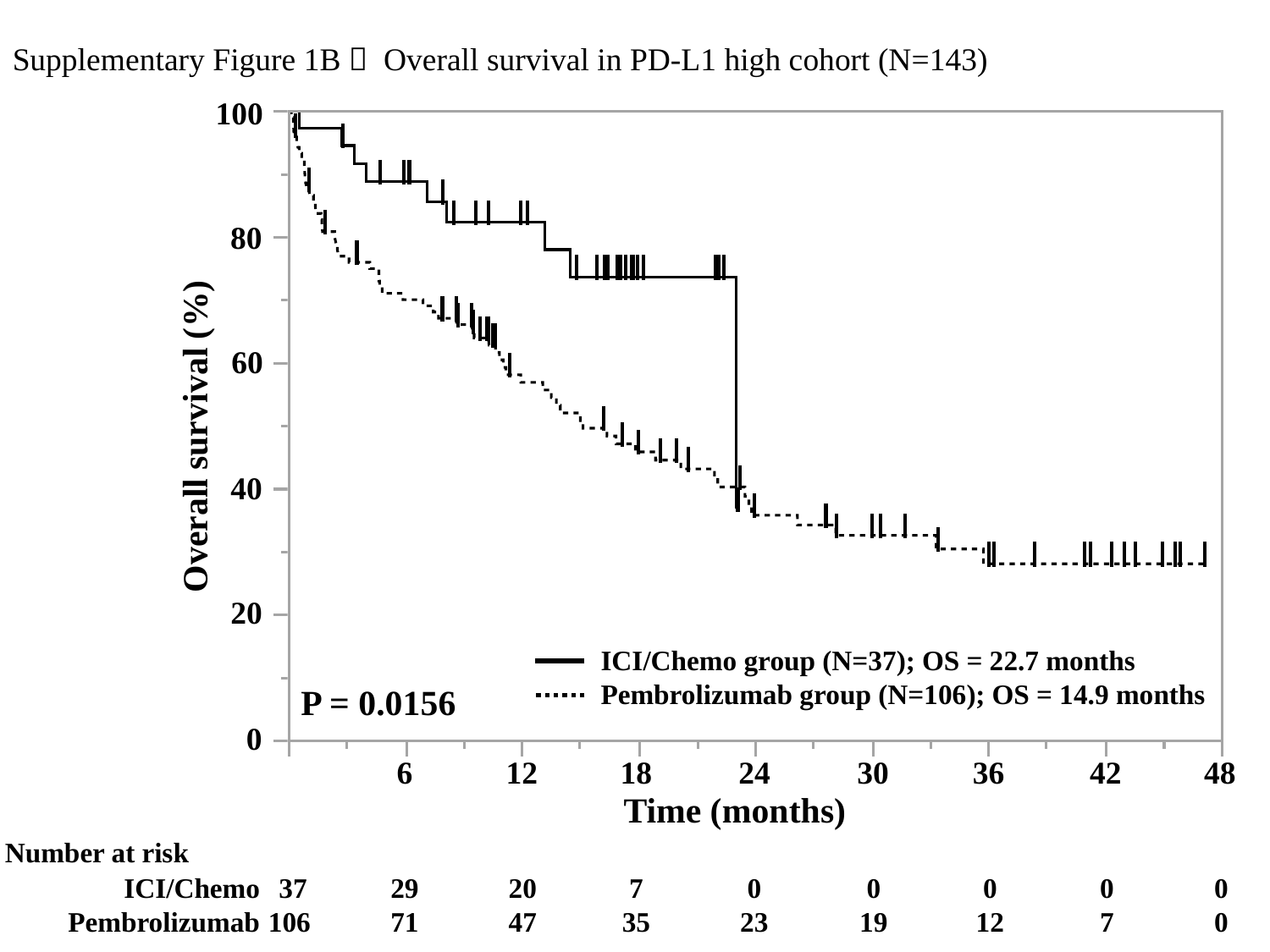

Supplementary Figure 1B： Overall survival in PD-L1 high cohort (N=143)
100
Overall survival (%)
80
60
40
20
ICI/Chemo group (N=37); OS = 22.7 months
Pembrolizumab group (N=106); OS = 14.9 months
P = 0.0156
0
6
12
18
24
30
36
42
48
Time (months)
Number at risk
ICI/Chemo
Pembrolizumab
 37
106
29
71
20
47
7
35
0
23
0
19
0
12
0
7
0
0
