# Supplementary material for: Efficacy of Immune Checkpoint Inhibitor With or Without Chemotherapy for Nonsquamous NSCLC With Malignant Pleural Effusion: A Retrospective Multicenter Cohort Study
Source: JTO Clin Res Rep. 2022 Jun 3;3(7):100355. doi: 10.1016/j.jtocrr.2022.100355 (PMC9234704; doi:10.1016/j.jtocrr.2022.100355)
Supplement: Supplementary Figure2 [file mmc7.pptx]

## Slide 1
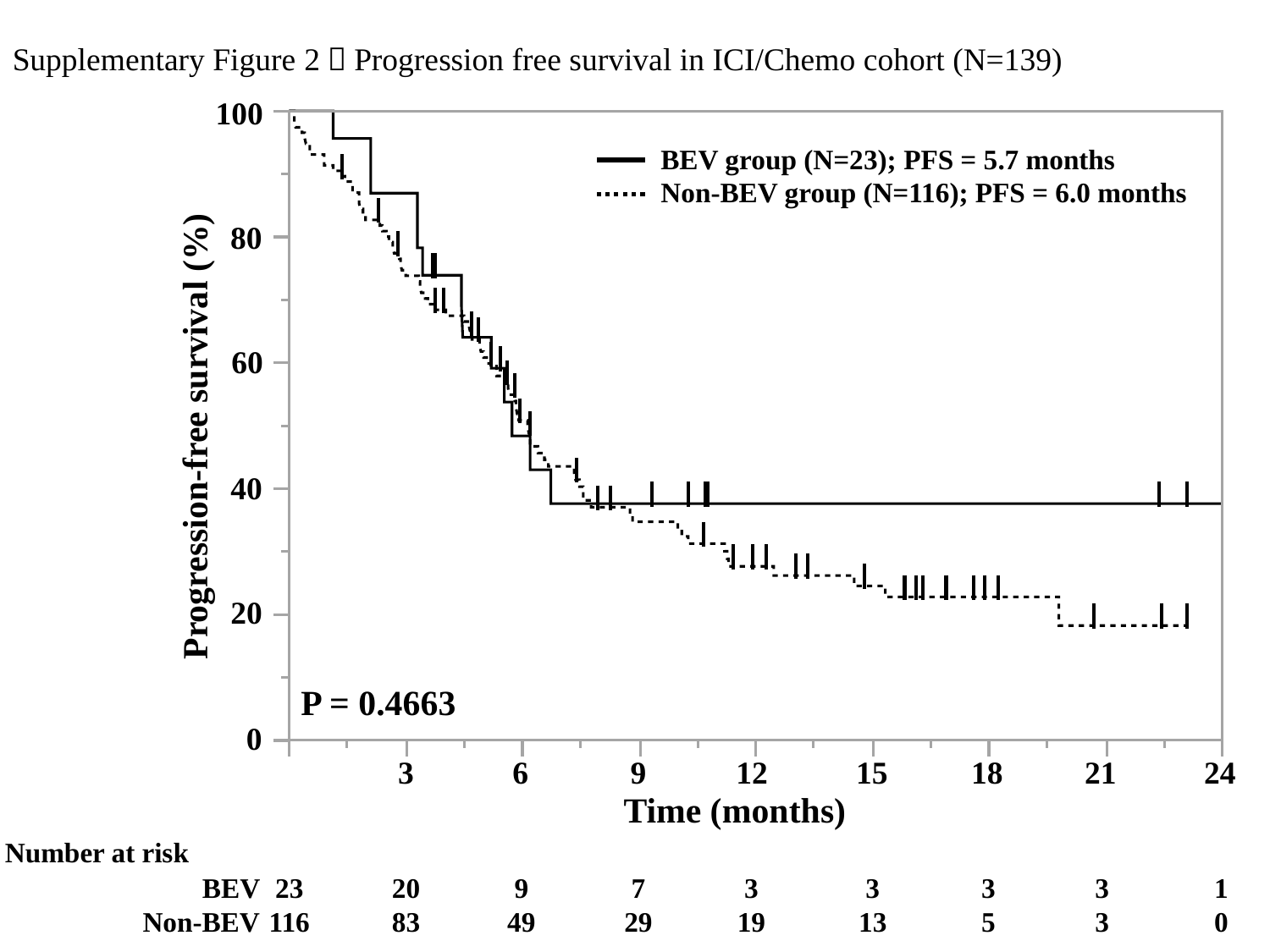

Supplementary Figure 2：Progression free survival in ICI/Chemo cohort (N=139)
100
BEV group (N=23); PFS = 5.7 months
Non-BEV group (N=116); PFS = 6.0 months
Progression-free survival (%)
80
60
40
20
P = 0.4663
0
3
6
9
12
15
18
21
24
Time (months)
Number at risk
BEV
Non-BEV
23
116
20
83
9
49
7
29
3
19
3
13
3
5
3
3
1
0
